# Supplementary material for: Crustal rejuvenation stabilised Earth’s first cratons
Source: Nat Commun. 2021 Jun 10;12:3535. doi: 10.1038/s41467-021-23805-6 (PMC8192532; doi:10.1038/s41467-021-23805-6)
Supplement: Supplementary file 1 — Supplementary Information [file 41467_2021_23805_MOESM1_ESM.pdf]

Supplementary Figure 1: Yilgarn Craton detrital zircon age distributions

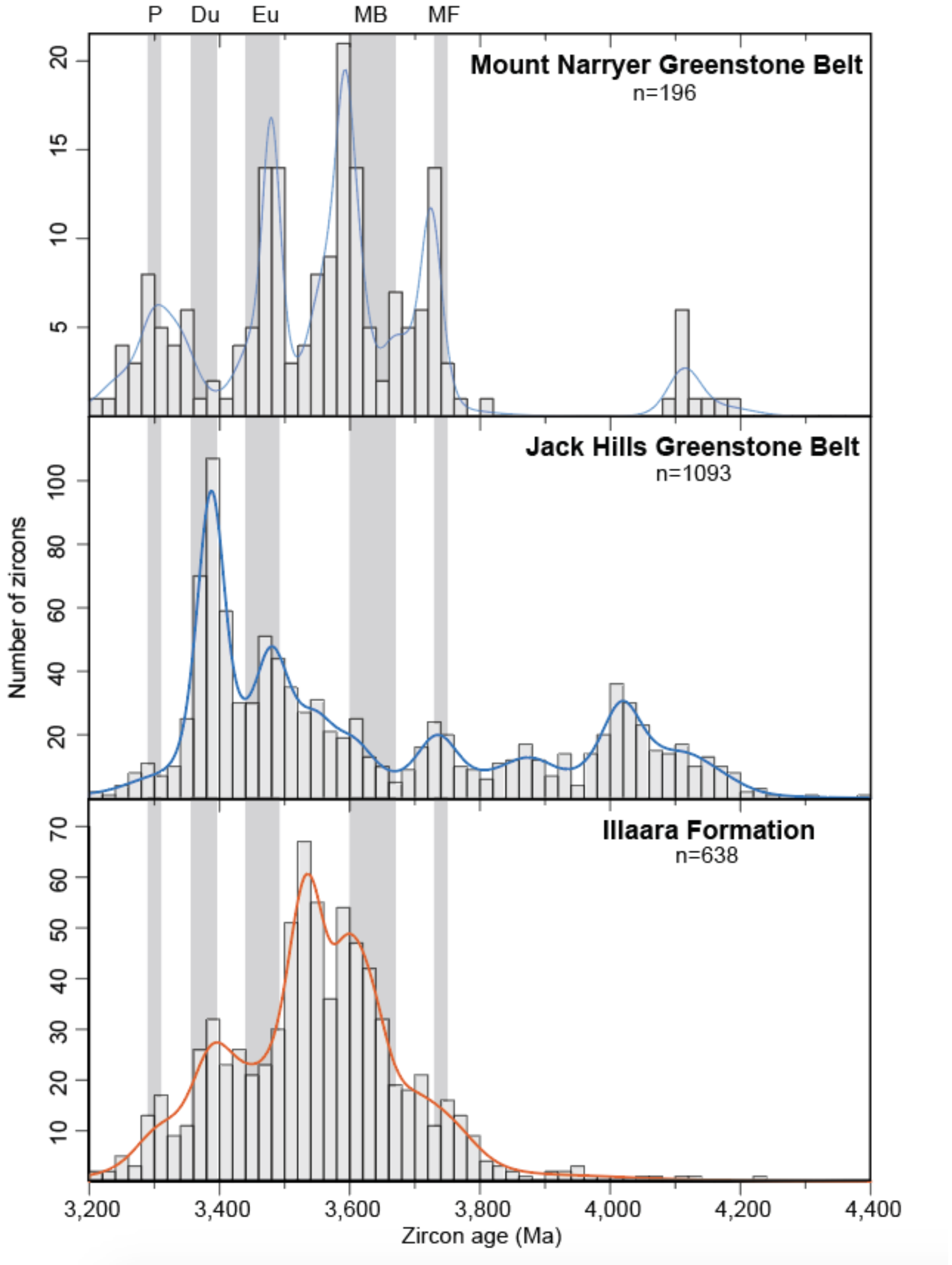

Supplementary Figure 1 | Kernel density estimations and histograms of detrital zircon ages from ~3,000 Ma metasedimentary successions in the Yilgarn Craton[1–7]. The grey bands behind the plots show the age range of igneous and metamorphic units exposed in the Narryer Terrane[8]: MF, Manfred Complex; MB, Meeberrie Gneiss; G, unnamed granodiorite; Eu, Eurada Gneiss; Du, Dugel Gneiss; P, late porphyritic granites. Compiled data sources listed below.

Supplementary Note 1: Compilation of zircon Hf isotopic composition from Eoarchaean Cratons

All compiled zircon $\varepsilon Hf_{(t)}$ are re-calculated at their published $^{207}Pb/^{206}Pb$ ages using the ^{176}Lu decay constant of ref. 9 and the CHUR parameters of ref. 10

Data from cratons showing a step-shift in zircon Hf isotope evolution coincident with onset of crustal preservation (Figure 3b) include:

Wyoming Craton

Detrital zircons from Mesoarchean quartz-rich strata in the Beartooth Mountains[11] and zircons from magmatic rocks in Sacawee block gneisses in the Beartooth-Bighorn Magmatic Zone[12].

The oldest exposed evolved crust in the Wyoming Craton comprises ~3,500 Ma TTG orthogneisses in the Bighorn-Beartooth magmatic zone[13]. Reworking of Hadean crust in the formation of the Wyoming Craton is inferred from Pb isotope systematics of late Archean granites[14,15].

Singhbhum Craton

The Singhbhum Craton compilation includes detrital zircons from modern river sediments[16] and the Mesoarchean Mahagiri Quartzite[17]. Magmatic and xenocrystic zircons from Older Metamorphic Tonalitic Gneiss[18] and Singhbhum Granite Complex[19] are also included in the compilation. The oldest preserved evolved crust in the Singhbhum comprises 3,530 Ma components of the Older Metamorphic Tonalitic Gneiss[18,19].

Slave Craton

The Slave Craton compilation includes zircons from igneous rocks in Acasta Gneiss Complex[20−23] and igneous[24] and detrital[25] zircons from the Central Slave Basement Complex. The Slave Craton is unique in containing extant Hadean evolved crust (the 4,020 Ma Idiwhaa Gneiss)[20], which together with multi-age component ~3,900—3,750 Ma evolved gneisses occur as small enclaves in a voluminous ~3,600 Ma granitoid complex[20−23]. We follow ref 24. in interpreting the emplacement of these ~3,600 Ma granitoids as marking the key episode of crustal stabilisation in the Slave Craton.

Yilgarn Craton

The Yilgarn Craton compilation uses the data plotted on figure 2 and includes data from detrital zircons from the Illaara Formation (this study) and detrital zircons from the Jack Hills metasedimentary belt that were analysed by concurrent the Pb-Hf[26,27]. The oldest evolved crust in the Yilgarn Craton comprises ~3,730 Ma TTG gneisses[8].

Zimbabwe Craton (not included in Figure 3b)

Although Hf isotope data from early Archean detrital zircons is available from Zimbabwe Craton and adjacent Limpopo Belt[28,29], the combined dataset does not define a clear Hf isotope step-shift and so was not included on Figure 3b. However, Bauer et al.[30] recently recognised a possible step-shift in zircon Hf isotopic compositions to juvenile sources at 3,600 Ma based only on the Zimbabwe Craton data. This possible isotopic shift coincides with the formation of the oldest crust in the craton (Supplementary Figure 2) and thus supports our interpretation of a link between crustal rejuvenation and crustal stabilisation.

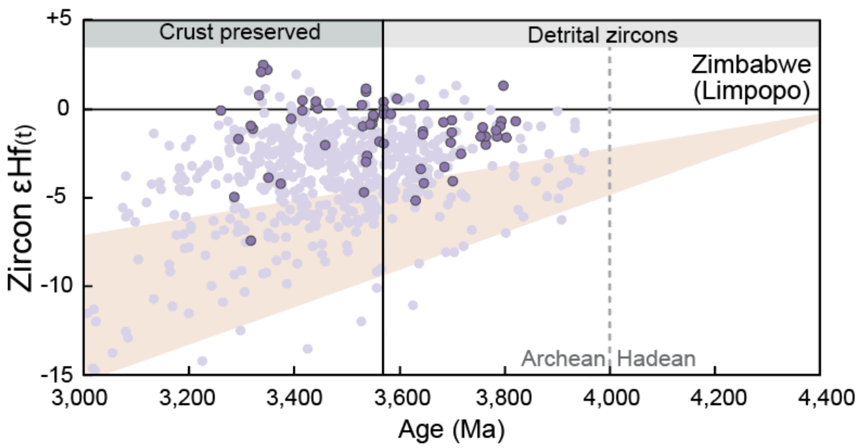

Supplementary Figure 2: Detrital zircon Hf isotope data for Zimbabwe Craton. Detrital zircons from the Zimbabwe Craton are shown as dark circles, whereas those from the Limpopo Belt are shown as light circles. A shift to juvenile Hf isotopic compositions at ~3,600 Ma observed in the Zimbabwe Craton data[30] overlaps temporally with the formation of the oldest preserved crust in the craton (the 3,565 Ma Towke gneisses).

The compilation of zircon Hf isotope data for the oldest crust in Eoarchean cratons (Figure 3c) and evidence for reworking of Hadean crust in their formation (Figure 3a) includes data from the following sources:

Ukaranian Shield

The oldest evolved crust in Ukrainian Shield for which zircon Hf isotope data are available is enderbite sample 06-BG38 from the Dniester-Bug Series in the Podolian Domain[31]. The enderbite complex in the Dniester-Bug Series is interpreted to include older components dating back to ca. 3,750 Ma (e.g., sample C10-U4 of ref. 31), however, zircon Hf isotope data are not available for these rocks. Claesson et al.[31] calculated the zircon $\varepsilon Hf_{(t)}$ for sample 06-BG38 at 3,750 Ma based on the interpretation that entire enderbite complex was emplaced at 3,750 Ma. However, given that many Eoarchean gneiss complexes are characterised by interleaving of rocks of vastly different ages at the outcrop scale, we adopt a more conservative approach and calculate zircon $\varepsilon Hf_{(t)}$ for 06-BG38 at 3,600 Ma based on the cluster of the oldest zircon core ages in this sample. Calculating the zircon $\varepsilon Hf_{(t)}$ for 06-BG38 at 3,750 Ma age as proposed by ref. 31 yields a value of +5, which is also consistent with our interpretation that oldest crust in Ukranian Shield involved an important contribution from a juvenile source.

São Francisco Craton

The oldest crust in the São Francisco Craton is exposed in the Mairi Gneiss Complex of the Gavião Block. We combined zircon Hf isotope data from the two oldest samples reported by ref. 32, which include sample 17ED-14.1 (a granodioritic gneiss) with a preferred age of 3642 Ma and sample 18DE-17 (a dioritic gneiss) with a preferred age of 3638 Ma. In addition to the negative zircon $\varepsilon Hf_{(t)}$ of the Eoarchean gneisses in the Mairi Gneiss Complex[32], independent evidence for the reworking of Hadean crust in the construction of the São Francisco Craton comes from a 4,100 Ma xenocryst from the Archean Ibitira-Ubiracaba greenstone belt in the Gavião Block[33].

Kaapvaal Craton

The Ancient Gneiss Complex of Swaziland hosts the oldest crust exposed in the Kaapvaal Craton. The oldest dated evolved rock in the Ancient Gneiss Complex for which zircon Hf isotope data is available is tonalitic gneiss sample AG6c-4c, which has a magmatic age of

3662 Ma[34]. The former presence of Hadean crust in Kaapvaal Craton is inferred from: (1) negative ^{142}Nd anomalies in granitoids and amphibolites from the Ancient Gneiss Complex and volcanic units in the Barberton greenstone belt[35] and (2) Hadean detrital zircons from the Barberton greenstone belt[36], however, no Hf isotope data are currently available for these zircons.

Tarim Craton

The oldest crust in the Tarim Craton comprises several exposures of ~3,720 Ma tonalitic gneiss in the Aktash Gneiss Complex[37,38]. Our compilation combines the zircon Hf isotope data for samples 16ALT67 and 16ALT68 reported by ref. 38, with zircon $\varepsilon Hf_{(t)}$ calculated at 3,720 Ma.

Yilgarn Craton

The oldest crust in the Yilgarn Craton includes the Meeberrie Gneiss—a multi-component gneiss of broadly TTG composition— and the predominantly mafic-ultramafic Manfred Complex, which both yield zircon U-Pb ages of ~3,730 Ma[39]. Our compilation includes Hf isotope data from the 'Set A' zircons of Meeberrie Gneiss sample 88-28 reported by ref. 40.

Superior Craton

The oldest evolved rocks in the Superior Craton are ~3,750 Ma TTGs in the Nuvvuagittuq greenstone belt[41]. Our compilation includes zircon data from sample PC-287 (a 3757 Ma trondhjemite band) and sample PC-286 (a 3,756 Ma TTG) from the southern portion of the Nuvvuagittuq greenstone belt[42]. Some granitoids and mafic-ultramafic units in the Northeastern Superior Craton have negative ^{142}Nd anomalies, which are inferred to reflect the reworking of Hadean crust[42,43].

North China Craton

The Anshan Complex hosts the oldest crust in the North China, which includes small outcrops of purported ~3,800 Ma TTGs in the Baijiafen, Dongshan, Guodishan and Shengousi complexes[44]. Our compilation includes zircon Hf isotope data from the 3802 Ma trondhjemitic gneiss sample 05FW032 from the Baijiagen Complex[45]. Hadean detrital zircons recovered from Paleozoic cover sequences in the North China Craton may reflect the former presence of Hadean crust in this area[44].

East Antarctic Craton

Eoarchean gneisses are known from several exposures in the Napier Complex of East Antarctica. These gneisses experience ultrahigh temperature metamorphism in the Neoarchean, which severely disturbed U-Pb systematics of many samples, resulting in large age uncertainties[46]. The oldest magmatic age thus far reported is from a sample of granitic orthogneiss from Gage Ridge (78285013). Harley and Kelley[46] combined previously published zircon data from 78285013 and calculated a preferred age of 3,877 ± 62 Ma, which is equivalent to the more recent estimate of 3,857 ± 39 Ma based on a smaller dataset reported by ref. 47. Our compilation includes the least radiogenic zircon Hf isotopic analyses from refs. 40 and 47, with $\varepsilon Hf_{(t)}$ calculated at 3,877 Ma. In addition to the negative zircon $\varepsilon Hf_{(t)}$ in sample 78285013, a ~3,850 Ma TTG from nearby Mt Sones yields a negative ^{142}Nd anomaly, consistent with involvement of Hadean crust in the formation of the Napier Complex[47].

North Atlantic Craton

The Itsaq Gneiss Complex in the Nuuk region of southern West Greenland contains several (meta-)tonalites yielding zircon ages in excess of 3,800 Ma. The 3,880 Ma meta-tonlite sample G01/36-1 reported by ref. 48 is taken here as the oldest dated rock in the North Atlantic Craton for the purposes of our compilation. Other comparably old (> 3,800 Ma) TTGs in the North Atlantic Craton yield similar, broadly chondritic zircon Hf isotope signatures to G01/36-1[49–51]. Reworking of Hadean crust in the formation of the Itsaq Gneiss Complex is reflected in the Pb isotopic systematics of orthogneisses and metasediments[52].

Supplementary Figure 3: Measured U-Pb ages for reference materials

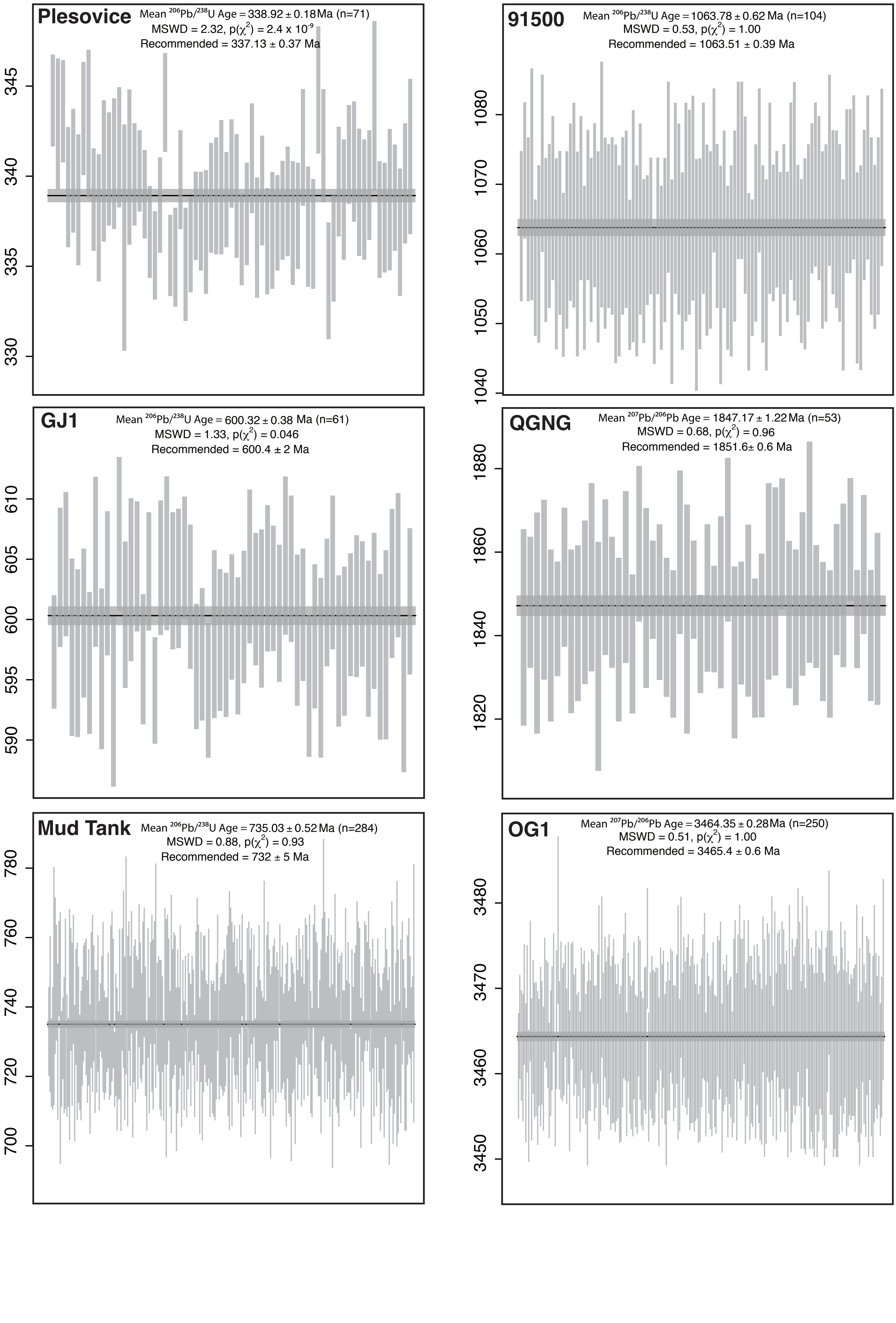

Supplementary Figure 4: Measured Hf isotope ratios for reference materials

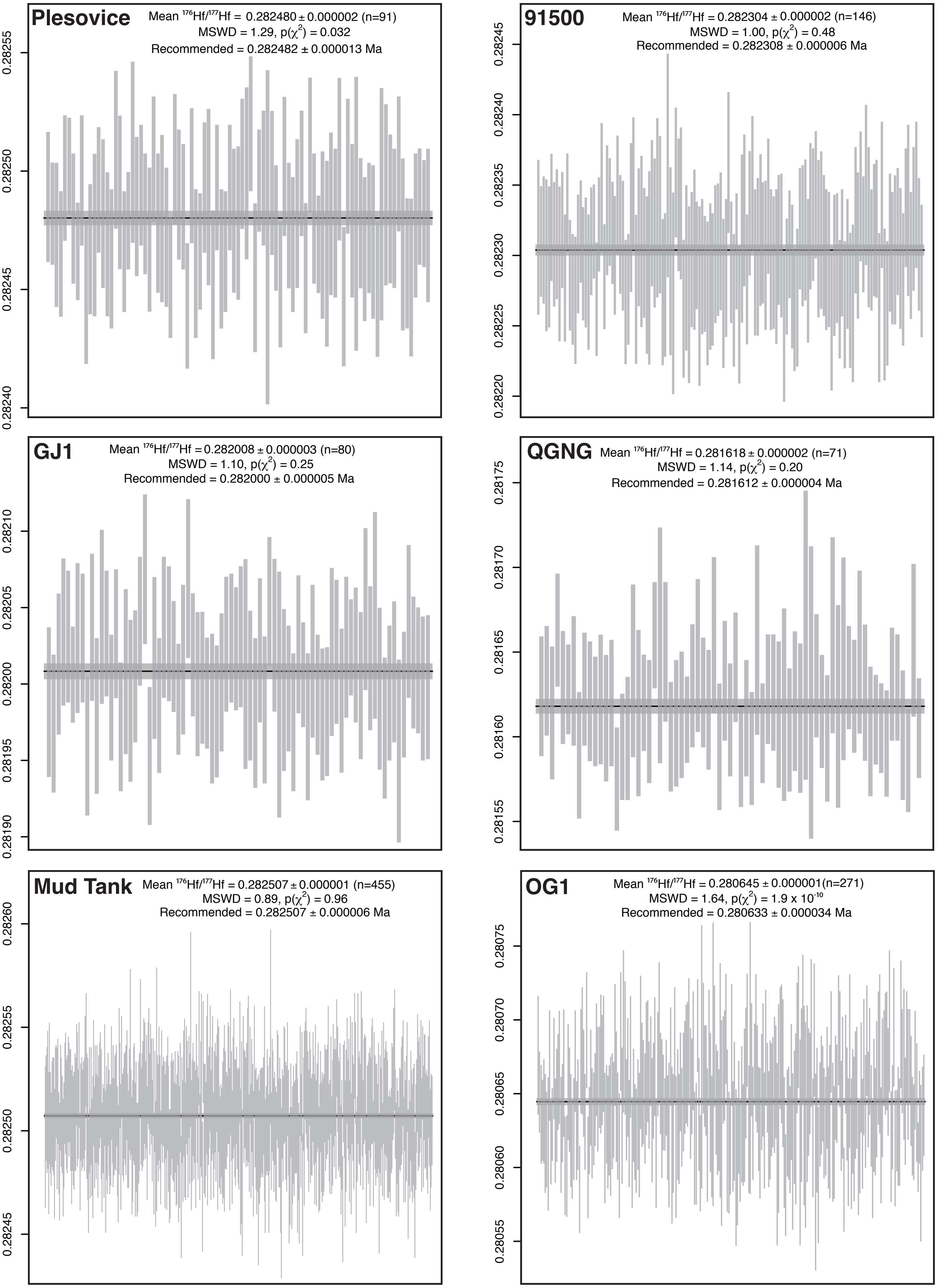

Stable Isotope Ratios

Mean $^{178}Hf/^{177}Hf$ = 1.467196 ± 0.0000008
Recommended = 1.467170 ± 0.00029

Mean $^{180}Hf/^{177}Hf$ = 1.886622 ± 0.0000018
Recommended = 1.886660 ± 0.00038

NB: There may be fewer analyses included in the U-Pb age calculations compared to the Hf isotope ratios for some reference materials as some analyses yielded acceptable Lu-Hf data but poor U-Pb data (the latter often due to analyses overlapping cracks, inclusions, or older ablation pits). Error bars are 2 sigma.

Supplementary references

1. Kinny, P. D. , Wijbrans, J. R., Froude, D.O., Williams, I.S., & Compston, W. Age constraints on the geological evolution of the Narryer Gneiss Complex, Western Australia, Australian Journal of Earth Sciences, **37**, 51—69. (1990)

2. Lu, Y, Wingate, MTD, Kirkland, CL, Goscombe, B & Wyche, S. 198644: pelitic gneiss, Mount Narryer; **Geochronology Record 1286:** Geological Survey of Western Australia, 6p. (2015).

3. Lu, Y, Wingate, MTD, Kirkland, CL, Goscombe, B and Wyche, S 2015, 198654: quartzite, Mount Narryer; **Geochronology Record 1287**: Geological Survey of Western Australia, 6p.

4. Lu, Y, Wingate, MTD, Kirkland, CL, Goscombe, B and Wyche, S 2015, 198669: pelitic gneiss, Mount Narryer; **Geochronology Record 1288:** Geological Survey of Western Australia, 6p.

5*. Wang, Q., & Wilde, S. A. New Constraints on the Hadean to Proterozoic history of the Jack Hills belt, Western Australia. *Gond. Res.* **55**, 74—91 (2018).

6. Cavoise, A. J., Valley, J. W., Wilde, S. A. *Earth's oldest rocks,* 2nd edn, *(*eds Van Kranendonk, M. J., Bennett, V. C., & Hoffmann, J. E) 239–253 (Elsevier, 2019).

7. Holden, P. et al. Mass-spectrometric mining of Hadean zircons by automated SHRIMP multi-collector and single-collector U/Pb zircon age dating: the first 100,000 grains. *Int. J. Mass Spectrom.* **286**, 53e63 (2009).

8. Kemp, A. I. S., Wilde, S. A., Spaggiari, C. *Earth's oldest rocks,* 2nd edn, *(*eds Van Kranendonk, M. J., Bennett, V. C., & Hoffmann, J. E) 239–253 (Elsevier, 2019).

*(See refs. 6 and 7 for a more comprehensive summary of age distribution of Jack Hills zircons. Here, we take the dataset of ref. 5 as representative of Jack Hills age distribution as the age peaks are similar to those observed in previously published datasets, but has the advantage of being collected in a single laboratory and used a typical analysis interval, unlike the U-Pb survey results presented in ref. 7).

9. Söderlund, U., Patchett, P.J., Vervoort, J. D., & Isachsen, C. E., The ^{176}Lu decay constant determined by Lu-Hf and U-Pb isotope systematics of Precambrian mafic intrusions. *Earth Planet. Sci. Lett.* **219**, 311–324. (2004)

10. Bouvier, A., Vervoort, J. D. & Patchett, P. J. The Lu–Hf and Sm–Nd isotopic composition of CHUR: Constraints from unequilibrated chondrites and implications for the bulk composition of terrestrial planets. *Earth Planet. Sci. Lett.* **273**, 48–57 (2008).

11. Mueller, P. A., & Wooden, P. A. Trace Element and Lu-Hf Systematics in Hadean-Archean Detrital Zircons: Implications for Crustal Evolution. *J. Geol.* **120**, 15–29 (2012).

12. Frost, C.D., et al. Hadean origins of Paleoarchean continental crust in the central Wyoming Province. *Geol. Soc. Am. Bull.* **129**, 259–280. (2017).

13. Mueller, P.A., Wooden, J.J., Mogk, D.W., Nutman, A.P., & Williams, I.S. Extended history of a 3.5 Ga trondhjemitic gneiss, Wyoming Province, USA: Evidence from U-Pb systematics in zircon: *Precambr. Res.* **78**, 41–52 (1996).

14. Wooden, J. L., & Mueller, P. A. Pb, Sr, and Nd isotopic compositions of a suite of late Archean igneous rocks, Eastern Beartooth Mountains – implications for crust–mantle evolution. *Earth Planet. Sci. Lett.* **87**, 59–72 (1988).

15. Kamber, B. S., The evolving nature of terrestrial crust from the Hadean, through the Archaean, into the Proterozoic. *Precambr. Res.* **258**, 48—82 (2015).

16. Miller, S. R., et al. Detrital zircons reveal evidence of Hadean crust in the Singhbhum Craton, India. *J. Geol.* **126**, 541–552 (2018).

17. Ranjan, S., Upadhyay, D., Pruseth, K. L., & Nanda, J. K. Detrital zircon evidence for change in geodynamic regime of continental crust formation 3.7—3.6 billion years ago. *Earth Planet. Sci. Lett.* **538**, 116206. (2020).

18. Chaudhuri, T., Wan, Y., Mazumder, R., Ma, M., & Liu, D. Evidence of Enriched, Hadean Mantle Reservoir from 4.2-4.0 Ga zircon xenocrysts from Paleoarchean TTGs of the Singhbhum Craton, Eastern India. *Sci. Rep.* **8**, 1—12 (2018).

19. Dey, S., Topno, A., Liu, Y., & Zong, K. Generation and evolution of Palaeoarchaean continental crust in the central part of the Singhbhum craton, eastern India. *Precambr. Res.* **298**, 268–291 (2017).

20. Reimink, J.R., et al. No evidence for Hadean continental crust within Earth's oldest evolved rock unit. *Nat. Geosci.* **9**, 777–780 (2016).

21. Guitreau, M., Blichert-Toft, J., Martin, H., & Mojzsis, S.J. Hafnium isotope evidence from Archean granitic rocks for deep-mantle origin of continental crust. *Earth Planet. Sci. Lett.* **337–338**, 211–223 (2012).

22. Bauer, A.M., Fisher, C.M., Vervoort, J.D., Bowring, S.A. Coupled zircon Lu–Hf and U–Pb isotopic analyses of the oldest terrestrial crust, the >4.03 Ga Acasta gneiss complex. *Earth Planet. Sci. Lett.* **458**, 37–48 (2017).

23. Iizuka, T. et al. Reworking of Hadean crust in the Acasta gneisses, northwestern Canada: Evidence from in-situ Lu-Hf isotope analysis of zircon. *Chem. Geol.* **259**, 230—239 (2009).

24. Reimink, J. R., Pearson, D. G., Shirey, S. B., Carlson, R. W., & Ketchum, J. W. F. Onset of new, progressive crustal growth in the central Slave craton at 3.55 Ga. *Geochem. Persp. Let.* **10**, 8–13 (2019).

25. Pietranik, A. B. et al. Episodic, mafic crust formation from 4.5 to 2.8 Ga: New evidence from detrital zircons, Slave craton, Canada. *Geology,* **36**, 875–878 (2008).

26. Kemp, A. I. S., et al. Hadean crustal evolution revisited: new constraints from Pb–Hf isotope systematics of the Jack Hills zircons. *Earth Planet. Sci. Lett.* **296**, 45–56 (2010).

27. Bell, E. A., Harrison, T. M., Kohl, I. E., & Young, E. D. Eoarchean crustal evolution of the Jack Hills zircon source and loss of Hadean crust. *Geochim. Cosmochim. Acta* **146**, 27–42 (2014).

28. Bolhar, R., et al. Juvenile crust formation in the Zimbabwe Craton deduced from the O-Hf isotopic record of 3.8–3.1 Ga detrital zircons. *Geochim. Cosmochim. Acta* **215**, 432–446 (2017).

29. Zeh, A., Stern, R. A., & Gerdes, A., The oldest zircons of Africa—Their U–Pb–Hf–O isotope and trace element systematics, and implications for Hadean to Archean crust–mantle evolution. *Precambr. Res.* **241**, 203–230 (2014).

30. Bauer, A. M., et al. Hafnium isotopes in zircons document the gradual onset of mobile-lid tectonics. *Geochem. Persp. Let.* **14**, 1—6 (2020).

31. Claesson, S., Bibikova, E., Shumlyanskyy, L., Dhuime, B., & Hawkesworth, C. J., 2015. Continent Formation Through Time. (eds. Roberts, N. M. W., Van Kranendonk, M., Parman, S., Shirey, S. & Clift, P. D.. Continent Formation Through Time. *Geol. Soc. Spec. Publ.* **389**, 227–259 (2015).

32. Oliveira, E. P., McNaughton, N. J., Zincone, S. A., & Talavera, C.,. Birthplace of the São Francisco Craton, Brazil: Evidence from 3.60 to 3.64 Ga Gneisses of the Mairi Gneiss Complex. *Terra Nova*, **2020;00,** 1—9 (2020).

33. Paquette, J.-L., et al. The geological roots of South America: 4.1 Ga and 3.7 Ga zircon crystals discovered in N.E. Brazil and N.W. Argentina. *Precambr. Res.* **271**, 49–55 (2015).

34. Zeh, A., Gerdes, A., & Millonig, L. Hafnium isotope record of the Ancient Gneiss Complex, Swaziland, southern Africa; evidence for Archaean crust–mantle formation and crust reworking between 3.66 and 2.73 Ga. *J. Geol. Soc. London*, **168**, 1–11 (2011).

35. Schneider, K. P., Hoffmann, J. E., Boyet, M., Munker, C., Kroner, A. Coexistence of enriched and modern-like ^{142}Nd signatures in Archean igneous rocks of the eastern Kaapvaal Craton, southern Africa. *Earth Planet. Sci. Lett.* **487**, 54–66 (2018).

36. Byerly, B. L., et al. Hadean zircon from a 3.3 Ga sandstone, Barberton greenstone belt, South Africa. *Geology,* **46**, 967–970 (2018).

37. Ge, R. F., Zhu, W.B., Wilde, S.A., & Wu, H.L. Remnants of Eoarchean continental crust derived from a subducted proto-arc. *Sci. Adv.* **4**, aao3159 (2018).

38. Ge, R. F, et al. Generation of Eoatchean continental crust from altered mafic rocks derived from a chondritic mantle: The ~3.72 Ga Aktash gneisses, Tarim Craton (NW China). *Earth Planet. Sci. Lett.* **538**, 116225 (2020).

39. Kemp, A. I. S. Early earth geodynamics: cross examining the geological testimony. *Philos. Trans. R. Soc. A* **376**, 20180169 (2018).

40. Hiess, J., & Bennett, V. C. Chondritic Lu-Hf in the early crust-mantle system as recorded by zircon populations from the oldest Eoarchean rocks of the Yilgarn Craton, West Australia and Enderby Land, Antarctica. *Chem. Geol.* **427**, 125–143 (2016).

41. O'Neil, J., Boyet, M., Carlson, R.W., & Paquette, J.-L., Half a billion years of re- working of Hadean mafic crust to produce the Nuvvuagittuq Eoarchean felsic crust. *Earth Planet. Sci. Lett.* **379**, 13–25 (2013).

42. O'Neil, J., Carlson, R.W., Francis, D., & Stevenson, R.K. Neodymium-142 evidence for Hadean mafic crust. *Science*, **32**, 1828–1831 (2008).

43. O'Neil, J., & Carlson, R. W. Building Archean cratons from Hadean mafic crust. *Science*, **355**, 1199–1202 (2017).

44. Wan, Y., et al. *Earth's oldest rocks,* 2nd edn, *(*eds Van Kranendonk, M. J., Bennett, V. C., & Hoffmann, J. E) 293—327 (Elsevier, 2019).

45. Wu, F.Y., Zhang, Y.B., Yang, J.H., Xie, L.W., & Yang, Y.H., Zircon U-Pb and Hf isotopic constraints on the early Archean crustal evolution in Anshan of the North China Craton. *Precambr. Res.* **167**, 2339—2362 (2008).

46. Harley, S.L., & Kelly, N.M., *Developments in Precambrian Geology* vol. 15. (eds. van Kranendonk, M. J., Smithies, H. R., Bennett, V. C.) 149–186 (Elsevier, 2007).

47. Guitreau, M., et al. Hadean protocrust reworking at the origin of the Archean Napier Complex (Antarctica). *Geochem. Persp. Let.* **12**, 7—11 (2019).

48. Hiess, J., Bennett, V. C., Nutman, A.P., & Williams, I.S. In situ U–Pb, O and Hf isotopic compositions of zircon and olivine from Eoarchaean rocks, West Greenland: New insights to making old crust. *Geochim. Cosmochim. Acta* **73,** 4489–4516 (2009).

49. Næraa, T., at al. Hafnium isotope evidence for a transition in the dynamics of continental growth 3.2 Gyr ago. *Nature* **485**, 627–630 (2012).

50. Fisher, C. M., & Vervoort, J. D. Using the magmatic record to constrain the growth of continental crust—the Eoarchean zircon Hf record of Greenland. *Earth Planet. Sci. Lett.* **488**, 79–91 (2018).

51. Vezinet, A., et al. Hydrothermally-altered mafic crust as source for early Earth TTG: Pb/Hf/O isotope and trace element evidence in zircon from TTG of the Eoarchean Saglek Block, N. Labrador. *Earth Planet. Sci. Lett.* v. **503**, 95–107 (2018).

52. Kamber, B. S., Collerson, K. D., Moorbath, S., Whitehouse, M. J. Inheritance of early Archaean Pb-isotope variability from long-lived Hadean protocrust. *Contrib. Mineral. Petr.*, **145**, 25–46 (2003).
