# Supplementary material for: Crustal rejuvenation stabilised Earth’s first cratons
Source: Nat Commun. 2021 Jun 10;12:3535. doi: 10.1038/s41467-021-23805-6 (PMC8192532; doi:10.1038/s41467-021-23805-6)
Supplement: Supplementary file 3 — Description of Additional Supplementary Files [file 41467_2021_23805_MOESM3_ESM.pdf]

# Description of Additional Supplementary Files

File name: Supplementary Data 1

Description: U-Pb-Hf isotopic data from detrital zircons from the Illaara Formation.
